# A Review on the Methods Used for the Detection and Diagnosis of Rabbit Hemorrhagic Disease Virus (RHDV)

**DOI:** 10.3390/microorganisms9050972

**Published:** 2021-04-30

**Authors:** Joana Abrantes, Ana M. Lopes

**Affiliations:** 1 CIBIO/InBio-UP, Centro de Investigação em Biodiversidade e Recursos Genéticos, Universidade do Porto, 4485-661 Vairão, Portugal; jabrantes@cibio.up.pt; 2 Departamento de Biologia, Faculdade de Ciências da Universidade do Porto, 4169-007 Porto, Portugal; 3 Instituto de Ciências Biomédicas Abel Salazar (ICBAS)/Unidade Multidisciplinar de Investigação Biomédica (UMIB), Universidade do Porto, 4050-313 Porto, Portugal

**Keywords:** rabbit hemorrhagic disease virus, detection, European rabbit

## Abstract

Since the early 1980s, the European rabbit (*Oryctolagus cuniculus*) has been threatened by the rabbit hemorrhagic disease (RHD). The disease is caused by a lagovirus of the family *Caliciviridae*, the rabbit hemorrhagic disease virus (RHDV). The need for detection, identification and further characterization of RHDV led to the development of several diagnostic tests. Owing to the lack of an appropriate cell culture system for in vitro propagation of the virus, much of the methods involved in these tests contributed to our current knowledge on RHD and RHDV and to the development of vaccines to contain the disease. Here, we provide a comprehensive review of the RHDV diagnostic tests used since the first RHD outbreak and that include molecular, histological and serological techniques, ranging from simpler tests initially used, such as the hemagglutination test, to the more recent and sophisticated high-throughput sequencing, along with an overview of their potential and their limitations.

## 1. Introduction

Rabbit hemorrhagic disease (RHD) is a highly contagious, acute and fulminating disease that affects primarily the European rabbit (*Oryctolagus cuniculus*) [1]. The disease was first described in 1984 in China [2], and dispersed worldwide within a few years [1]. In some countries, such as Australia, RHD was introduced as a biocontrol agent [3]. RHD is caused by rabbit hemorrhagic disease virus (RHDV), a lagovirus of the family *Caliciviridae*. Transmission occurs by the oral, nasal or conjunctival routes [4]. The incubation period of RHD varies between one and three days, and clinical signs often include respiratory and neurological disorders, as well as apathy and anorexia. Upon necropsy, the most common lesions are found in the liver (hepatic necrosis), spleen (splenomegaly), lungs and trachea (hyperemia and hemorrhages). While the liver is mainly used in diagnostic methods for viral identification, as it contains the highest viral titers, the spleen might also be of interest, especially when chronic or subacute forms of the disease are present [4]. Nonetheless, RHDV can be detected in other organs and tissues, such as lung, kidney and bone marrow [1,5], but also in biological fluids such as serum, urine and feces [6,7].

RHDV is a single-stranded, positive-sense RNA virus, with a genome of ~7.4 kb and a 3′ poly-A tail [8]. The virus has two open reading frames (ORFs), with ORF1, after translation and proteolytic cleavage, producing the non-structural proteins and the major capsid protein, VP60, and ORF2 being responsible for a minor structural protein, VP10 [9]. A subgenomic RNA of ~2.2 kb is also found in RHDV virions and is the major source of VP60 protein [10]. RHDV virions are non-enveloped, spherical particles, with a diameter between 27–35 nm and the typical caliciviruses’ cup-shaped depressions [11]. Due to its high stability in the environment, RHDV can be detected in degraded tissue samples, even under harsh environmental conditions, with no need for preservation in stabilizers such as RNA later, e.g., [12], and in tissues kept frozen for several years, e.g., [13,14].

Phylogenetically, four RHDV genotypes have been identified [15]. GI.1, often referred to as the classical form, groups the first strains characterized worldwide [16,17,18] and was the only pathogenic genotype circulating for more than 20 years. Four variants are recognized within GI.1, GI.1a-d [16], with GI.1a having distinct antigenic properties and being mostly associated with outbreaks in rabbitries [19]. GI.2 is a new genotype that emerged in France in 2010 [20] and was responsible for massive declines in the European rabbit populations [21]. Interestingly, while GI.2 is able to cause death in young rabbits (<two months old) and hare species, such as the *Lepus europaeus*, *L. timidus*, *L. corsicanus* and *L. capensis* [22,23,24,25], there are no reports of massive GI.1 outbreaks affecting hares and kittens. The large divergence (>15%) between GI.1 and GI.2 might be responsible for the incomplete/low protection of vaccines developed against GI.1 in GI.2 outbreaks [20]. Finally, genotypes GI.3 and GI.4 correspond to the non-pathogenic forms of RHDV [26,27]. In the 2000s, with the advances in the sequencing capacity of genetic laboratories, several intra and intergenotype recombination events started to be described [28,29]. Importantly, GI.2 origin and evolution seems to be closely associated with recombination at the polymerase/capsid junction [30,31,32].

RHDV continues to represent an important threat to the European rabbit and the related industry, with significant ecological and economic disturbances, e.g., [33]. A tentative diagnosis can be made based on the epidemiological features, clinical signs and characteristic pathological changes, but they might overlap with other rabbit-affecting diseases, such as pasteurellosis, clostridial enterotoxaemia and rabbit pox [2,34,35]. This led to the development of several diagnostic methods and their standardization by the World Organisation for Animal Health (Office International des Epizooties, OIE) reference laboratory for RHD. Research groups working on RHDV have been using different diagnostic methods that include electron microscopy, immunosorbent assays, PCR and high-throughput sequencing, among others, or a combination of methods. Much of the methods were developed from the lack of an in vitro culture system for RHDV. However, the extent of diversity found in lagoviruses, with patterns of cross-reactivity between RHDV variants and genotypes in antibody-based methods, and the intricate epidemiological picture, with co-circulation of different viruses in natural populations and in rabbitries, make the decision on the best suitable diagnostic method rather challenging. Thus, this review provides a compilation of the applicability, sensitivity, and specificity of each method along with the pros and cons of their use. We intend to provide a glimpse on the state-of-the-art of each method in order to allow researchers an informed decision on the best approach according to their needs.

## 2. Hemagglutination Test

Due to the non-cultivable nature of RHDV, the hemagglutination test (HA) was among the first RHDV diagnostic methods [2]. The HA is based on the ability of RHDV to strongly agglutinate type 0 human red blood cells (RBCs), which was already documented in the first description of the disease in 1984, as the result of binding to glycolipid ligands present on the RBCs’ surface [2]. It was later demonstrated that RHDV agglutination activity was similar for human RBCs belonging to type 0, A, B and AB blood groups [36]. This culminated in the identification of ABH blood group antigens as attachment factors used by RHDV to initiate infection [37]. ABH antigens are present in RBCs from adult humans, but absent from RBCs from other mammalian species [38], which explains the lower or null RHDV hemagglutination activity in RBCs from other species, e.g., [2,39,40].

The HA is performed as a micromethod and takes advantage of small sample volumes or highly diluted tissue suspensions. The human type 0 RBCs used for testing the agglutination activity of the viral strains must be freshly collected. RBCs are then washed and resuspended in phosphate buffered saline (PBS) [4]. Liver or spleen samples are prepared as clarified tissue homogenates [4]. Equal volumes of washed RBCs and liver or spleen samples are used [4]. Samples exhibiting agglutination at an endpoint dilution greater than 1/160 are considered RHDV-positive; samples with titers <1/160 should be re-tested using other methods to confirm that they are indeed RHDV-negative [4]. RHDV HA titers are detected in the liver as early as 2 h post-infection and rapidly rise to a maximum that is reached at the time of the rabbit’s death (10 × 2^14^ to 10 × 2^16^) [41]. On average, titers range from 10 × 2^12^ to 10 × 2^18^ in liver, spleen and serum samples of RHDV-infected rabbits [42], but lower to almost null HA titers are found in the lungs, kidney, heart, lymph nodes, muscles and brain [39,43,44], probably reflecting the low viral concentrations in these organs. Rabbits showing subacute/chronic forms of RHD might also test negative [45], although this is dependent on the characteristics of the virus particles [4]. Temperature has been shown to affect the HA activity of some RHDV strains [4]. Repeated freezing and thawing of the samples significantly alter HA titers [2] while cold storage, even for long periods, has no effect [42].

The need for limited amounts of sample, the low cost and simplicity are among the major advantages of the HA. However, the requirement for human blood to test the agglutination activity, which is difficult to obtain due to the biological risk of its manipulation, presents a major limitation to the use of the HA. In addition, the low sensitivity and specificity and the need for non-standard conditions for some RHDV strains are further limitations of the test. Indeed, both false negative and false positive HA results are documented for RHDV. False positive samples might be due to the presence of other microorganisms with hemagglutination activity, such as *Pasteurella* and parvovirus [42]. As for the false negatives, low antigen concentration, the presence of degraded virus particles from inadequate sample processing and storage and a chronic course of disease are among possible causes [42,46]. Additionally, a few RHDV strains have been shown to present non-hemagglutination activity, e.g., [42,47,48,49,50,51,52] or the hemagglutination activity is only seen when conditions differ from the standard protocol (e.g., the RHDV Rainham strain, GenBank accession number AJ006019 only hemagglutinates at 4 °C) [53]. Finally, the HA does not allow the distinction between variants nor genotypes [54]. Despite the development of more sensitive and robust techniques, the HA is still widely used in some countries for preliminary diagnosis of RHDV, and was even recently validated in the diagnostic of GI.2 [54,55]. Its ability to detect non-pathogenic strains (genotypes GI.3 and GI.4) remains to be determined.

## 3. Electron Microscopy

**Figure 1:**
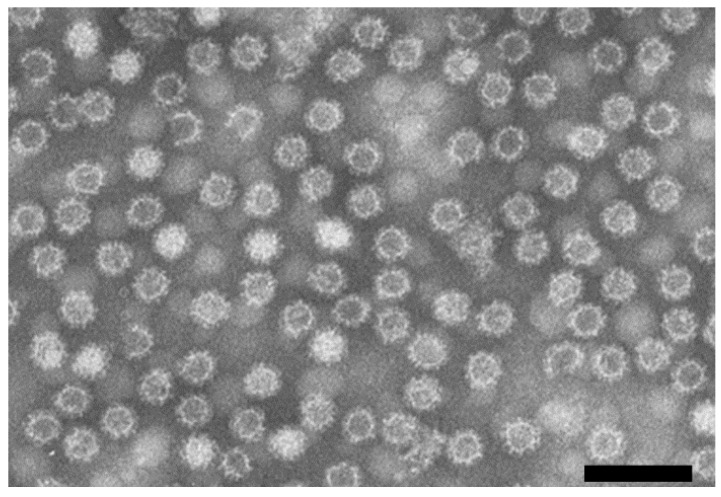
Electron microscopy (EM) microphotograph of purified RHDV (GI.1) particles isolated from the liver of an infected rabbit. Particles show the cup-shaped depressions that are typical of the calicivirus morphology. Scale bar = 100 nm. Image kindly provided by Dr. Antonio Lavazza and Dr. Lorenzo Capucci, IZSLER, Italy.

The early identification of RHDV as a calicivirus relied on electron microscopy (EM) examination, with virus particles presenting the typical arranged cup-shaped depressions of this family, along with a size of ~35 nm, and lacking an envelope (Figure 1) [56,57]. Furthermore, when rabbits in Europe started to die with clinical signs similar to those described in China, Valicek and colleagues showed by immunoelectron microscopy (IEM) the antigenic relationship between Chinese and European strains [57].

For EM, tissues must be mechanically homogenized and centrifuged to clear the supernatant as for HA; ultracentrifugation is advisable to concentrate the viral particles and increase sensitivity. As the liver, lung, kidney and spleen have the highest RHDV titers, diagnosis by EM relies on these tissues [58]. Different techniques can be applied prior to visualization in the electron microscope. In negative-staining EM, a drop of the homogenate is placed on a formvar/carbon-coated grid and virions will appear translucent against a dark background (Figure 1), thanks to the use of a negative stain solution, the most commonly used being phosphotungstic acid (PTA) [59]. The low sensitivity and specificity of the method might be enhanced by using immune EM (IEM) for diagnostic purposes, which combines the more traditional approach with the use of monoclonal antibodies (MAbs) produced against RHDV or hyperimmune sera from RHDV-infected rabbits [4].

This simple and rapid technique has been also used for research purposes, not only to characterize the causative agent of disease, e.g., [60,61], but also to confirm infection after experimental inoculations, e.g., [62,63,64] and to assess the reliability and correct self-assembly of virus-like particles (VLPs), e.g., [65,66,67,68], which is particularly useful for vaccine production. Electron microscopy was critical in the identification of smooth (s-RHDV) particles associated with chronic infections [45]. These particles, with morphology and diameter different from that of RHDV, derive from intact virions with dissociated protrusion, i.e., correspond only to the inner shell of RHDV [69]. The most up-to-date, high-resolution models of the RHDV capsid were reconstructed at 6.5–11 Å with cryo-electron microscopy [69,70,71], a powerful method capable to disentangle more complex structures [72].

In the last decades, advances in viral diagnosis had been in part due to EM techniques, mostly because it does not need previous knowledge of the virus and sample preparation requirements are not difficult to fulfill. However, virus identification is based solely on morphology, requires high amounts of viral particles (around 10^5^ viral particles/mL) [59], and needs expensive equipment/maintenance and an experienced technician. As an alternative, researchers may hire external services to perform EM with full technical support. Although currently less used, EM is still a highly valuable diagnostic tool, especially if combined with other testing methods. Nevertheless, this technique might not be doable when screening large numbers of samples and might not be appropriate to discriminate RHDV variants.

## 4. Immunohistochemistry

**Figure 2:**
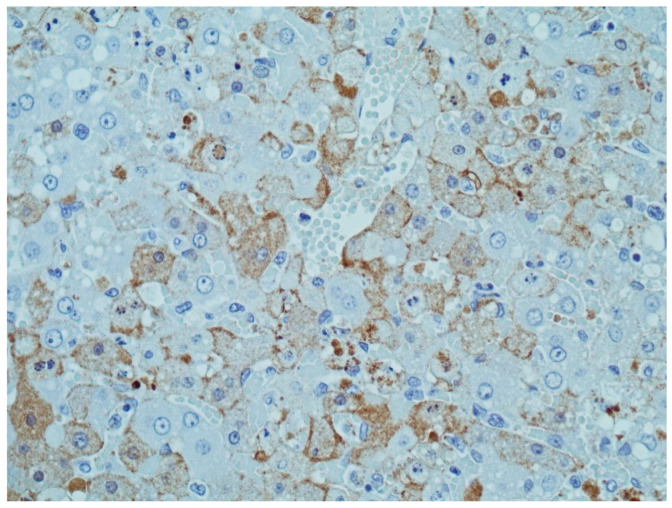
Photomicrograph of immunohistochemistry applied to the liver from a rabbit infected with GI.2. Brown staining indicates the presence of the viral capsid antigen in numerous hepatocytes. 400× magnification. Image kindly provided by Dr. Aleksija Neimanis, SVA, Sweden.

Immunohistochemistry (IHC) is a technique of immunostaining, i.e., an antibody-based method of detection of RHDV, which involves the identification of RHDV antigens in tissue sections fixed in 10% buffered formalin and embedded in paraffin. In some countries, it has been used as a complementary diagnostic tool to confirm RHDV infection [60,73] and, more recently, for a full characterization of GI.2 and its distribution in adult and young rabbit tissues (Figure 2) [6]. IHC has also been used for purposes other than the simple detection of RHDV [74,75].

IHC protocol starts with deparaffinization and rehydration of tissues in successive ethanol baths. Then, endogenous peroxidase activity is blocked with hydrogen peroxide and non-specific binding is blocked with a bovine serum albumin (BSA) containing buffer to limit background interference. Slides are then incubated with a monoclonal or polyclonal anti-RHDV antibody. The most commonly used systems in IHC for RHDV detection are the avidin-biotin-complex (ABC)-peroxidase [62,76,77] and the streptavidin-biotin-peroxidase [78,79].

The use of IHC in RHDV diagnosis was also a step further in the knowledge of RHD progression. Indeed, IHC allows to monitor the in situ localization of RHDV in the tissues and to correlate it with histopathological findings [6]. RHDV is usually detected in degenerative and necrotic hepatocytes with intense intranuclear staining and diffuse intracytoplasmic staining, suggestive of virus replication in these cells [62,76,80,81]. These hepatocytes localize predominantly in the periportal areas and, to a lesser extent, in midzonal areas, indicating antigen spread as the disease progresses [6,76,81]. Positivity in these cells can be detected as early as 12 h post-infection [77,81], even though other studies refer 24 h [80] or 40 h post-infection [78]. By IHC, the virus was also detected in Kupffer’s cells and endothelial cells of the liver [78], as well as in macrophages in the lungs, spleen, lymph nodes and bone marrow, indicating viremia [6,76]. An association between cells undergoing apoptosis and viral antigens’ distribution has also been established [78,79]. When infected with GI.1, young rabbits mount an early inflammatory reaction against RHDV, consistent with the finding by IHC that RHDV-infected hepatocytes appear close to inflammatory infiltrates [80,81,82]. Likewise, subacute forms of RHD present a patchy labelling of macrophages nearby inflammation areas [81].

The main advantages of IHC are the relatively low cost of the technique, its simplicity when comparing to IEM, the reasonably fast turnaround time and the possibility to retrospectively study old formalin-fixed paraffin-embedded tissues as well as fresh or frozen tissue samples [76], which was especially important when the first outbreaks of RHD were recorded. In addition, if the antibodies used are known to be specific for an RHDV genotype/variant and do not exhibit cross-reactivity, its identification might be possible. On the other hand, IHC needs an initial investment of equipment, there is a lack of a standardized procedure between laboratories, is prone to human error since it needs well-trained personnel to observe and interpret the results, and provides a qualitative rather than a quantitative diagnosis. Yet, and despite not being possible to quantify viral particles, the severity of histopathological lesions is indicative of the degree of infection [80]. In terms of detection limit, IHC is comparable to HA and to histopathological observations [76], but has lower sensitivity than in situ hybridization [83] and PCR. Estimations point to detection limits of 10^7^–10^8^ viral copies/mg of tissue [6].

Similar to IHC, but using fluorophores instead to visualize RHDV distribution in tissues and cells, immunofluorescence (IF) has been proposed as a routine diagnostic test due to its relatively easiness and rapidity to perform [42]. IF uses a MAb coupled to a fluorophore (direct fluorescence) or a secondary antibody conjugated to a fluorophore (indirect fluorescence). According to the OIE, specific fluorescence is detected in the liver, spleen and renal glomeruli [4]. IF has been used mainly for confirming expression of recombinant VP60 protein in infected cells [84,85,86]. Other authors used this technique to confirm virus specificity of hybridomas [87,88,89]. A typical IF protocol consists in the fixation of cells with cold acetone or ethanol, followed by incubations with a MAb and a FITC-conjugated secondary antibody. Expression of recombinant protein is examined for specific fluorescence using a fluorescence microscope. Distinction of genotypes/variants might be achieved using specific MAbs.

## 5. In Situ Hybridization

In the late 1990s, in situ hybridization (ISH) was developed as a method to detect RHDV RNA in formaldehyde-fixed, paraffin embedded tissue samples [83]. For that, non-radioactive digoxigenin (DIG)-labelled probes were used, which hybridized with the RHDV RNA in a region corresponding to nucleotide positions 4955 to 7437, thus including the entire capsid gene [83]. Antisense and sense DIG-labelled probes were developed, with a stronger signal provided by the antisense probe as it targeted the genome of replicated virions [83].

Briefly, the technique requires the generation of probes by cloning a fragment of RHDV genome that is usually the VP60 gene. Probe design is key to determine the ability of detecting more than one variant/genotype and must take into account virus evolution, as probes might lose sensitivity. Nonetheless, there are currently hundreds of RHDV VP60 sequences publicly available, and probe design in highly conserved regions diminishes this associated risk. After cloning, probes are prepared by in vitro transcription and DIG labelling with an RNA labelling kit. Dehydrated tissue sections are incubated with probes and hybridized probes are detected with anti-DIG antibodies. Temperature conditions and incubation times might vary [83,90,91].

The results obtained from ISH were considered reliable, highly specific and reproducible, with higher sensitivity than IHC [83]. ISH was applied in the study of RHDV pathogenesis [83], and later employed for determining the distribution of RHDV RNA in experimentally infected rabbits, which demonstrated that viral replication also occurs in macrophages [90], and for the tissue localization of Australian non-pathogenic strains [91]. However, as ISH implies high costs and a complicated methodology, its use as a method for RHDV diagnosis has been limited.

### Other Labelled Probes-Based Detection Methods

Labelled probes, either with DIG or radioactive compounds, were also combined with molecular methods used for the detection of RHDV to improve sensitivity and specificity [26,92,93,94]. One of the approaches consisted in southern blot hybridization where RHDV DNA amplified by reverse transcription polymerase chain reaction (RT-PCR) and detected by electrophoresis was transferred to a membrane and detected by RHDV-specific DIG or radio-labelled probes [92,93,94]. The other approach was based on the incorporation of DIG-labelled dUTPs during PCR amplification of a fragment of the capsid gene of RHDV [26]. Detection of the PCR products was then achieved with a colorimetric ELISA assay using a biotinylated hybridization capture probe derived from a conserved region within the amplified fragment (nucleotide positions 5922–5951) [26]. This detection method presented increased sensitivity when comparing with detection by conventional electrophoresis [26] and, with an adequate probe, it might allow the distinction of RHDV variants and genotypes.

## 6. Western Blot

**Figure 3:**
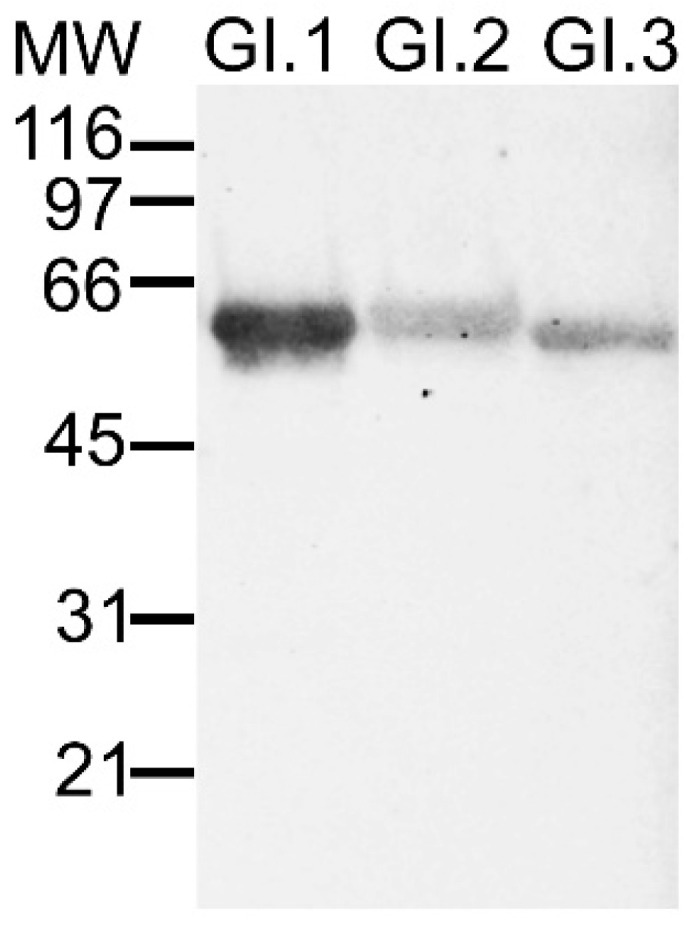
Western blot analysis of purified RHDV virus-like particles (VLPs) using an anti-RHDV serum obtained from a rabbit infected with GI.1. The serum recognizes the homologous GI.1 VLPs better than the heterologous GI.2 and GI.3 VLPs. Positions of molecular weight (MW) markers are shown in the left in kilodaltons. Image kindly provided by Dr. Esther Blanco and Dr. Juan Bárcena, INIA, Spain.

Western blotting (WB) or immunoblot is a commonly used technique to identify specific proteins. Regarding RHDV, the OIE recommends its use either when HA or ELISA are inconclusive or the samples contain s-RHDV particles [4]. It was used as a complementary diagnostic tool in several studies [62,63] and might also be applied to detect anti-RHDV antibodies present in rabbit sera [95]. In the first years after RHDV emergence, WB was a valuable molecular tool to identify structural proteins and determine their molecular weight (~60 kDa for VP60 and ~10 kDa for VP10; Figure 3) [9,56,96]. In WB, the minor structural protein VP10 has a weaker signal than VP60, reflecting its presence in smaller amounts [9]. Other bands of variable molecular weight might appear as a result of VP60 proteolytic degradation [42] or, in rabbits with subacute or chronic forms of the disease, as a result of the presence of s-RHDV [4]. Due to the lack of the cup-shaped depressions, s-RHDV particles have a molecular weight of ~30 kDa [45]. At the time of emergence of new genotypes, such as the non-pathogenic GI.3 and GI.4 and, more recently, GI.2, WB was used to confirm the identity of the capsid proteins with that of RHDV [26,27,55]. Currently, its major applications rely on the validation of recombinant proteins’ correct size (VLPs; Figure 3), e.g., [61,65,85,86,97,98], mapping epitopes recognized by MAbs [88] and confirming MAbs specificity and suitability [99].

For carrying out WB, sample homogenization and centrifugation are performed as described for EM, with an additional sucrose cushion ultracentrifugation step. To unfold the protein and enable access to the epitope, samples are denatured by heating in the presence of an anionic denaturing detergent. Proteins are then separated by molecular weight via a polyacrylamide gel electrophoresis and transferred by electroblotting to a solid membrane support (PVDF or nitrocellulose). To prevent non-specific background binding, the membrane is blocked with BSA or non-fat milk diluted, for example, in PBS or blocking buffer. The binding of specific antibodies, either MAbs or polyclonal antibodies, to the protein of interest follows and visualization is achieved due to labelling of the primary or secondary antibody (direct or indirect detection) [4]. Despite being more time-consuming, indirect detection is more sensitive. The higher sensitivity and specificity of WB when compared to the previous techniques relies on the separation of proteins by size, charge and conformation, and on the antibody-antigen interaction, a combination that makes it possible to detect even very low amounts of the protein of interest [100] and to distinguish RHDV genotypes/variants. Its major limitations are the need of a purified capsid protein or a specific anti-VP60 antibody, its non-quantitative nature, the requirement of technical training, the inaccuracy of sample preparation and the associated technical variance [100]. Yet, an accurate experimental setup can attenuate some of these limitations [100].

## 7. Hemagglutination Inhibition Test

The hemagglutination inhibition test (HIT) was the first serological test for the diagnosis of RHDV [2]. It detects and measures anti-RHDV antibodies by taking advantage of the hemagglutination ability of RHDV [40,42], but without RHDV genotype or variant differentiation. Serum samples are tested using human type 0 RBCs and titrated RHDV antigen.

For the HIT, the RHDV antigen is prepared from infected rabbit livers collected immediately upon death as described for HA. The supernatant is collected, filtered and titrated with the HA. Rabbit serum samples are heat inactivated and treated with kaolin followed by centrifugation. A second kaolin treatment is performed to improve specificity, which considerably increases the time to test each serum and limits the number of samples to be tested [42]. Serum samples are clarified by a final centrifugation step. The HIT is performed as a micromethod and uses a limited volume of the serum samples. Samples are serially diluted and incubated with the RHDV antigen containing a certain amount of HA units (between 4–8 as determined by the HA). Human type 0 RBCs are then added to each well. The hemagglutination inhibition titer is given by the last serum dilution showing complete inhibition of the hemagglutination. The positive threshold of serum titers is correlated to the titer of the negative control sera and usually ranges between 1/20–1/80.

Similar to the HA, the HIT by itself is a relatively cheap and easy laboratory technique that uses limited sample volumes. It was shown to be quite sensitive for the detection of anti-RHDV antibodies produced early in the infection [87]. However, the production of inconsistent results such as false positives [42,87] and the difficulty in accessing to human blood and the biological risks of its use led to its replacement by other ELISA-based techniques for diagnostic purposes [4] as these are quicker and easier, especially when there is a large number of samples to be tested, and because these have, in general, a higher sensitivity and specificity [4]. In addition, obtaining the RHDV antigen for the assay may not be straightforward with high costs as it implies an experimental infection and tissue processing to obtain a purified virus. Alternatively, VLPs can be produced by expressing a recombinant capsid protein in the baculovirus/Sf9 cell expression system which self-assemble to form particles structurally and antigenically identical to RHDV virions [65]. Yet, VLP production requires technical knowledge and equipment that usually does not exist in a standard molecular biology laboratory.

## 8. Enzyme-Linked Immunosorbent Assay

The enzyme-linked immunosorbent assay (ELISA) is a laboratorial test based on the use of antibodies and a colorimetric reaction to identify the presence of a certain antibody or antigen. The major benefits of ELISA are the cost-effectiveness, the easiness of data interpretation and the possibility to scale to testing large numbers of samples. Comparing with the HA, ELISA has higher sensitivity and specificity [42,47]. Depending on the setup employed, ELISAs may be qualitative—identifying a specific antigen or antibody present in the sample, quantitative—if including standard curves of known antigens, or semi-quantitative—comparison of the relative levels of antigen/antibody in the assay, considering that the intensity of signal is directly proportional to the antibody/antigen concentration. Blank controls must always be included in ELISA to discard background signal and artifacts of the experimental setup; additionally, when available, negative and positive controls should be included, e.g., sera from RHDV-susceptible rabbits and from vaccinated or convalescent animals, respectively [4].

Studies on RHDV using ELISA were performed for the detection of RHDV antigen and for the detection of anti-RHDV antibodies. ELISA was shown to be an accurate and rapid RHDV diagnostic tool already in 1989 [58]. The first evidence of the circulation of non-pathogenic rabbit caliciviruses came from serology testing by ELISA [26,96,101,102,103,104,105]. Non-pathogenic rabbit caliciviruses added further difficulty in assessing correct RHD diagnosis, as elicited serological responses might confound the results [4]. ELISA testing was also important for understanding RHDV epidemiology, by showing that species other than lagomorphs present humoral responses against RHDV. Indeed, non-target species as foxes, cats, stoats, ferrets, hedgehogs, hawks and gulls have been shown to naturally present anti-RHDV antibodies although in low titers [106,107,108].

### 8.1. Antigen Capture ELISA

Antigen-capture ELISA (ELISA-Ag) is generally used for detection of RHDV antigen, either by “sandwich” ELISA or variants of this technique [4]. ELISA development and implementation was a major breakthrough in the field of RHDV, as monitoring of the virus and RHD control depend on the knowledge of the circulating strains and their interactions [109]. ELISA-Ag assays are highly specific, sensitive and reliable, being able to detect the presence of RHDV in crude liver and spleen homogenates, but also in other tissues or organs, such as blood, serum and heart [89]. Subtyping of RHDV isolates can be achieved with a MAb-based ELISA developed by the OIE Reference Laboratory for RHD that also accounts for the more recent GI.2 [4,42], and several authors used this method for veterinary diagnosis [19,23,54,110,111,112,113]. Some variations of the basic procedure followed, such as the GI.2-specific ELISA developed by Dalton et al. [109], and the ELISA developed by the Australian Animal Health Laboratory after RHDV introduction in Australia as a biocontrol agent [89], which Australian researchers have been using ever since [114,115,116]. This ELISA alternatively uses a sheep anti-RHDV antibody as capture antibody. Additionally, commercial kits for the detection of RHDV antigen are available [74,117,118,119].

Briefly, the “sandwich” ELISA protocol for RHDV detection consists in coating a plate with high adsorption capability with an anti-RHDV hyperimmune serum. The use of a polyclonal hyperimmune anti-RHDV serum allows the identification of potential new variants. Tissue homogenates are added to the plate, followed by a horseradish peroxidase (HRP)-conjugated MAb. Given the high diversity found in lagoviruses, the OIE recommends the use of a panel of MAbs with different specificity [4]. The plate is then incubated with the chromogenic substrate. Samples must be run in duplicates and are considered positive if mean absorbance is 0.3 OD units greater than negative wells.

### 8.2. Antibody ELISA

While RHD diagnosis is pertinent to identify and manage outbreaks, the assessment of herd immunity is of extreme importance for rabbit management, as humoral response has a considerable effect in protecting animals from RHDV infection [96,120]. Indeed, characterization and titration of antibodies in vaccinated or convalescent rabbits might foresee higher ability to resist to an infection [4]. Having this in mind, serosurveillance studies were conducted to assess rabbit populations’ serostatus [121,122].

Protective immunity against RHD is reliably evaluated with competitive ELISA (cELISA) [120]. cELISAs have been developed by several groups working on RHDV [42,87,115] and are widely used [19,26,54,114,123,124,125,126]. Importantly, this technique not only identifies anti-RHDV antibodies but also antibodies developed against non-pathogenic lagoviruses [26]. The recommended OIE protocol for cELISA briefly consists in coating a specific polyclonal serum with high anti-RHDV titer in a high adsorption capability plate. Then, the serum sample and an antigen are added to the plate, followed by a HRP-conjugated rabbit immunoglobulin G (IgG) anti-RHDV; then the colorimetric reaction follows. A positive serum decreases by >25% the absorbance value of the reference value. As mentioned, and although HIT is more sensitive for early detection of antibodies, cELISA is quicker, highly specific, more consistent and requires less volume of serum [87].

Other types of ELISA were developed for diagnostic and research purposes, including blocking ELISA, “sandwich” ELISA, solid-phase ELISA (SP-ELISA) and indirect ELISA (iELISA). Blocking ELISA is a robust, specific and easy-to-perform ELISA for the detection of anti-GI.4 antibodies [127]. It is performed by coating a polyclonal antibody against GI.4, followed by the addition of a VLP; rabbit serum follows, then a mouse monoclonal antibody and finally an anti-mouse IgG. The main advantage of blocking ELISA, when compared to cELISA, is the two-step incubation of serum and MAb, which is more convenient and less time-consuming for large datasets [127].

iELISA working mechanism is similar to that of “sandwich” ELISA described above, but a VLP replaces the tissue homogenate, and serum binding to the VLP is recognized by a secondary antibody. SP-ELISA is simpler as it uses an antigen adsorbed to the plate to capture antibodies present in serum. As internal epitopes become exposed due to virus deformation, SP-ELISA has a wider sensitivity and lower specificity. Depending on the research aims, these might be fair alternatives to cELISA. cELISA has been shown to have improved specificity than iELISA, and overcame the difficulty of testing non-target animal species, as it does not need species-specific anti-Ig conjugates [87]. On contrary, if detection of cross-reactive antibodies is important for the purpose of the study, iELISA is more suitable [4]. Commercial kits for antibody detection, relying on iELISA, are available, for example, from Ingenasa, Noack, Clin-Tech and Abbkine.

Although there is a correlation between a positive antibody titer and protection against RHDV [4,46], in some cases it might be important for the interpretation of field serology to distinguish between different types of antibodies. As a result, isotype ELISAs (isoELISAs) have been developed to classify rabbits’ immunological status that enable the detection and titration of IgA, IgM and IgG antibodies [115,125], later adapted also to the non-pathogenic lagoviruses [128]. The former, although with higher background, avoids the need for blood samples and is a simple method to determine specific IgA levels [128]. IsoELISA results allow the discrimination of different conditions, such as antibodies resultant from past vs. recent infections, maternal antibodies or cross-reactive antibodies [115]. IgG is detected in serum by adsorbing a MAb in a plate, followed by the virus. Antibodies present in serum will bind to this complex and will be recognized by an anti-rabbit IgG HRP conjugate. For IgM and IgA detection, the protocol is slightly modified to avoid competition with IgG; therefore, anti-rabbit IgM or IgA are adsorbed to the wells and a RHDV specific HRP-conjugated MAb is used to detect isotypes [4,115]. Importantly, the different protocols do not allow the comparison of titers, i.e., equivalent titers do not correspond to same amounts of isotypes [115]. Positive sera must have an OD of >0.2 OD units above the negative control, and the last dilution giving a positive value is taken as the titer of that serum [4].

## 9. Reverse Transcription Polymerase Chain Reaction

The first reports of the use of PCR in the diagnosis of animal viral diseases date back to the late 1980s [129], concomitant with the worldwide dispersal of RHDV [1]. In 1991, the first complete RHDV genome was obtained with a combination of PCR and molecular cloning [8,130]. The molecular data obtained was then used for the determination of oligonucleotides (or primer sets) for the amplification of the 5′ region of the capsid gene of RHDV by RT-PCR [92]. These primers, which allowed the detection of as few as 12 copies of the template, confirmed the higher sensitivity of RT-PCR for the detection of RHDV, especially in comparison with the other tests existing at that time, and led to its establishment as a routine diagnostic test for RHDV [92]. Indeed, RT-PCR is 10^4^ times more sensitive than ELISA and HA, and is able to detect HA-negative RHDV strains [49,92,131]; it is also of rapid execution, with the possibility of testing several samples at the same time, can be highly specific and provides fast results.

Several types of tissues might be used to test for the presence of RHDV by RT-PCR. The liver, which is the main site of virus replication [90], is most commonly used, but kidney, spleen, lung, heart, brain, bone marrow, muscle, spinal cord, thymus and lymph perform well for the detection of RHDV [131,132]. Non-invasive or minimally-invasive samples such as nasal secretions, urine, feces and sera have also been successfully used [46,131].

RT-PCR requires RNA extraction from limited amounts of tissue samples (e.g., up to 50 mg of tissue samples, 200 µL of 10% (*w*/*v*) of tissue exudates or 250 µL of body fluids) and it can be carried out either as a one-step or two-step procedure. In the one-step RT-PCR, reverse-transcription, i.e., the cDNA synthesis from the viral RNA, and PCR amplification are performed in a single reaction vessel. This decreases contamination risk and significantly reduces hands-on time when comparing with the two-step procedure. However, since both reverse transcription and PCR reactions are conducted in a single tube, conditions for each reaction cannot be optimized separately. This can lead to lower yields or efficiency. In addition, the cDNA generated from the reverse transcription reaction cannot be used in subsequent reactions. In the two-step procedure, reactions are undertaken independently; the viral RNA is first reverse-transcribed into cDNA using gene-specific primers (GSP), oligo-d(T) and/or random hexamers and then used as a template for the PCR. The OIE Reference Laboratory for RHD recommends a single-step RT-PCR with a set of primers designed to amplify all GI.1 variants and GI.2 [4]. These primers target conserved regions of the capsid gene avoiding primer mismatches that could lead to unsuccessful amplification and hence, false-negative results [4]. 

**Figure 4:**
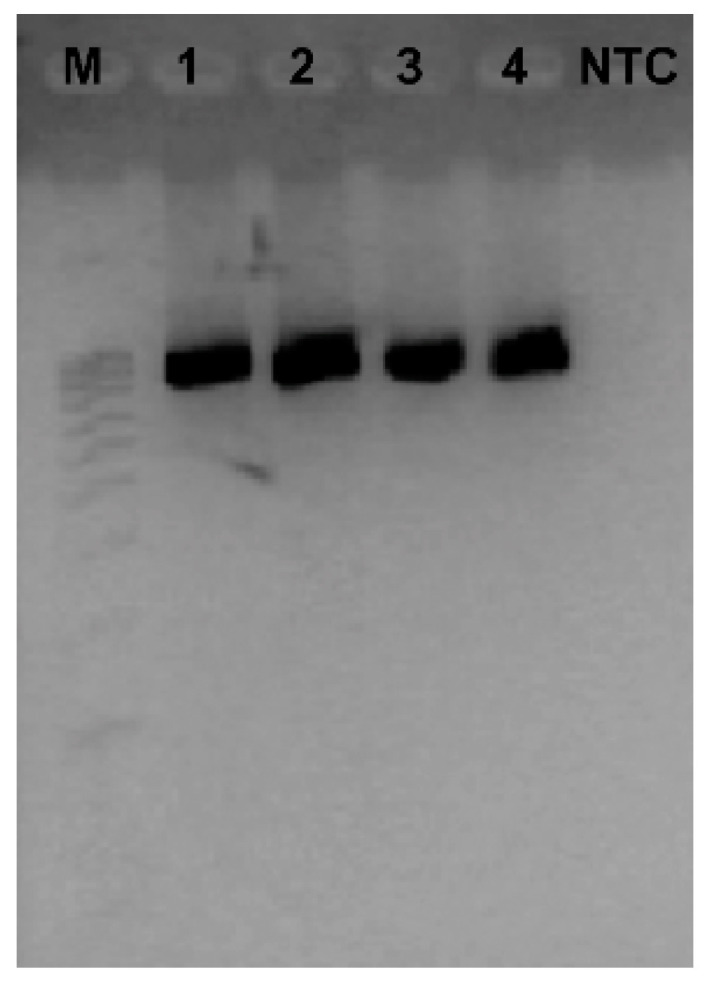
Detection of the partial VP60 amplification (nucleotides 6136–7121) of RHDV in rabbit liver samples by RT-PCR. M: molecular weight marker (Ladder V, Nzytech); lanes 1–4: rabbit samples infected with GI.2 strains; NTC: no template control.

Different protocols and primer sets, including primers that target other regions of the RHDV genome, e.g., [133], have been adopted by different research groups working on RHDV. Primer sets for the detection by RT-PCR of GI.2 (and its recombinants) and non-pathogenic GI.3 and GI.4 strains have also been developed, e.g., [26,27,54,55,134]. Detection of the correct amplification of the target sequence in the samples is made by gel electrophoresis (Figure 4) and confirmation of the specificity of the RT-PCR products can be further achieved by Sanger sequencing.

Variants of the RT-PCR for the detection of RHDV include nested RT-PCR (nRT-PCR), multiplex RT-PCR, reverse-transcription loop-mediated isothermal amplification (RT-LAMP) and immunocapture RT-PCR. In the nRT-PCR, two primer sets are used, with the second set, the nested primers, annealing within the fragment amplified by the first set and producing a shorter final PCR product. For RHDV, nRT-PCR was shown to increase both the specificity and sensitivity of the amplification reaction [135,136] and was first applied for testing Australian vertebrates for the presence of RHDV [137]. In addition, it was successfully used for RHDV detection from formalin-fixed, paraffin-embedded archived tissues further allowing retrospective studies [135]. However, nRT-PCR is more time-consuming and requires more a priori information on the target sequence than conventional RT-PCR [138].

A multiplex RT-PCR has been developed for the detection of the four pathogenic lagoviruses circulating in Australia [31]. Multiplex RT-PCR assays simultaneously detect more than one target in a single reaction, with different primer pairs specific for each target. This greatly reduces the number of reactions required, the amount of sample to be used and pipetting errors. Primers should be designed in regions highly conserved within variants but dissimilar between them [31], have similar melting temperatures and produce amplicons of different size. Variant/genotype assignment is based on gel electrophoresis run with no need to sequence PCR products. The main weakness of this approach is the preferential amplification of certain targets [139], which gives limited effectiveness in detecting mixed infections [31], and the requirement of a good knowledge of the sequence of the strains to be detected. Doubtful results should therefore be re-run in singleplex RT-PCR.

The RT-LAMP is a single-tube procedure that, following viral RNA extraction and one-step reverse transcription, amplifies a target DNA sequence under isothermal conditions [140,141]. The LAMP method principle is complex and more details can be found in [142,143]. For RHDV, it was optimized using four specific primer sets targeting the VP60 gene and was shown to be a simple and rapid method, with high specificity and sensitivity [144]. Indeed, RT-LAMP is 10^2^ times more sensitive than RT-PCR, but comparable to RT-qPCR [144]. It is also an alternative for limited-budget laboratories as it does not require any specialized equipment, but an isothermal water bath, and positive results can be easily detected by electrophoresis appearing as a ladder-like pattern in the gel or as a fluorescence emitting signal under UV light due to SYBR Green I staining [144]. While RT-LAMP seemed a promising method for the detection of RHDV, it has not been tested in strains other than Chinese [138] and requires a good knowledge on the sequence strains in order to design reliable primer sets.

In immunocapture RT-PCR (IC-RT-PCR), capture of the viral particles is performed via specific antibodies immobilized on a microplate, and viral RNA is released after enzymatic disruption. The RT-PCR follows using cDNA synthesized from the antibody-captured viral RNA and all the steps are carried out in a single reaction vessel [145]. As for RHDV detection, IC-RT-PCR was developed to overcome the hard-working and time-consuming sample preparation and nucleic acid extraction steps which hampered testing large amounts of samples [146]. Indeed, IC-RT-PCR considerably reduces the sample preparation time and allows the screening of more samples on a single day in comparison to previous methods such as HA, IEM and ELISA [146]. Specificity of this method relies on the interaction between the virus and the antibodies and between the PCR primers and the template [146]. IC-RT-PCR presents good specificity and a sensitivity 10^1^–10^2^ times greater than “sandwich” ELISA with no divergent results between them [146]. However, IC-RT-PCR requires a properly equipped laboratory, and reagents tend to be more expensive [146].

RT-PCR represented a major breakthrough to our knowledge on RHDV and RHD by allowing: (1) the comparison of sequences of RHDV strains from different geographic locations and collected in different years, e.g., [147], (2) the identification and molecular characterization of novel lagoviruses, including the non-pathogenic forms GI.3 and GI.4, the antigenic variant GI.1a, and more recently, the new genotype GI.2, and study their phylogenetic relationships, e.g., [19,26,27,54,148], (3) to test non-specific hosts and vectors with a possible role in the epidemiology of RHD, e.g., [137,149], (4) to detect spillover events, e.g., [14,23], (5) to monitor RHDV infection in different young and adult rabbit tissues, e.g., [150,151] and, (6) to study the mechanisms of RHDV evolution, and hypothesize about the possible origin of lagoviruses, e.g., [29,30,32,152,153]. However, the intricate epidemiological picture of lagoviruses, with co-circulation of pathogenic and non-pathogenic forms and/or variants, contributed to the development and optimization of other more sensitive and robust PCR-based techniques such as real-time quantitative RT-PCR.

## 10. Real-Time Quantitative RT-PCR

The real-time RT-PCR technology uses fluorescent dyes or probes that yield increased fluorescence with increasing amounts of dsDNA. Whilst conventional RT-PCR results are end-point, here specialized thermal cyclers monitor the fluorescence signal as amplification occurs, which is proportional to the amount of amplicon produced in each cycle. Real-time RT-PCR can either be semi-quantitative or quantitative (RT-qPCR). Several in-house protocols have been developed for the use of real-time RT-PCR, from genotype-specific to multiplex detection and quantification of lagoviruses, each adapted to the needs and epidemiological background of the region, e.g., [7,27,31,134,154,155]. A comprehensive review on such methods has been recently reported by Kwit and Rzeżutka (2019) [138]. Commercial kits are also available from, for example, Ingenetix and Genesig.

**Figure 5:**
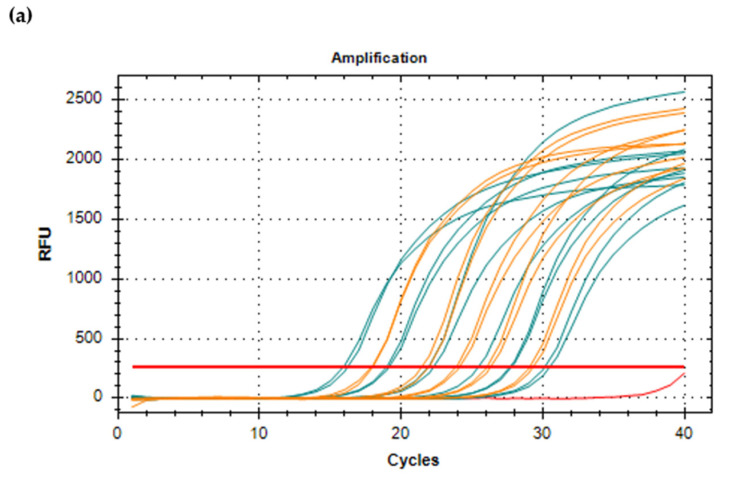

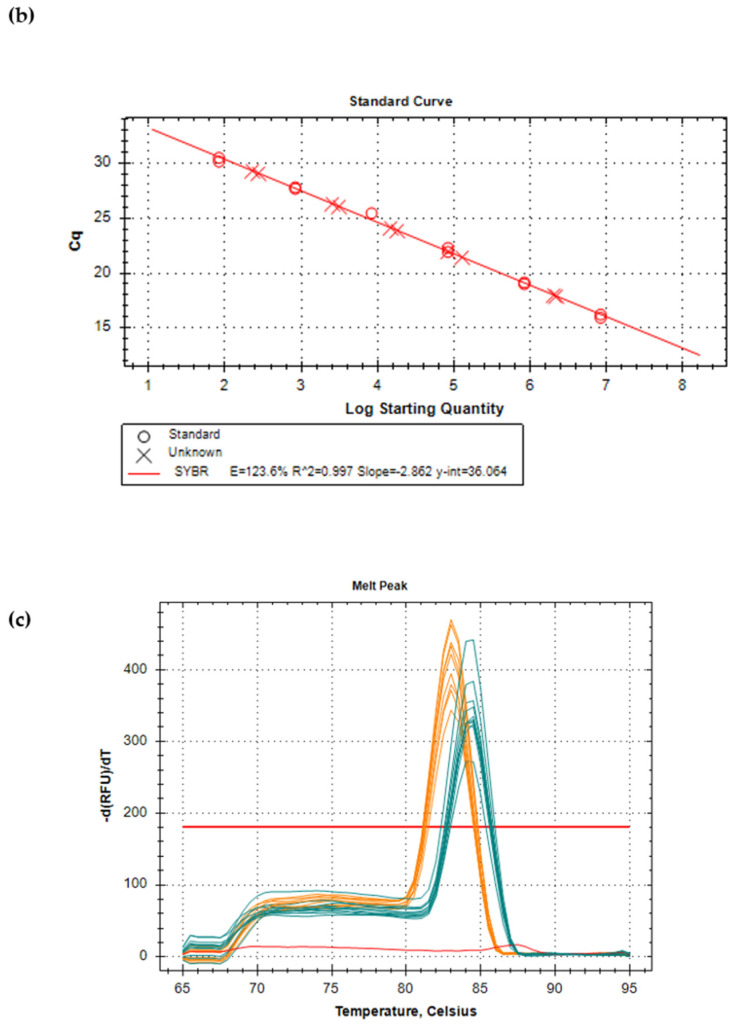
SYBR Green assay for quantification of viral RNA copies of RHDV in rabbit liver samples by RT-qPCR. Standards appear in blue, samples with unknown concentration appear in orange and no template controls appear in red. (**a**) Amplification curve; the starting quantity of the standard ranged from 8.35 × 10^6^ to 8.35 × 10^1^ copies/µL, (**b**) Standard curve; the log-basis for the *x*-axis in the regression line is 10, (**c**) melting curve. RFU: relative fluorescence units; −d(RFU)/dT: negative derivative of the fluorescence signal as a function of temperature; Cq: cycle threshold.

In order to perform a RT-qPCR, standards with known concentration/number of viral copies must be prepared by cloning a fragment of the RHDV genome (Figure 5a). For one-step RT-qPCR, after plasmid linearization, runoff transcripts, i.e., transcripts free of vector sequences, are produced with DNase treatment, and presence of residual DNA might be checked by conventional RT-PCR [156]. When selecting two-step RT-qPCR, cDNA synthesis is first performed independently in a regular thermal cycler. Primers and probes are designed in highly conserved regions, usually targeting the VP60. The reaction might include internal, endogenous controls to evaluate efficiency of RNA extraction, ascertain successful amplification and detect PCR inhibition, avoiding false negative results [7,27,134]. PCR inhibition is indeed observed in RNA obtained from several tissues, but a 1:10 dilution of the samples is enough to overcome such limitation [27]. Ideally, samples should be prepared as organ suspensions and run in duplicates. Absolute quantification of RNA molecule copies is then calculated from the standard curve (Figure 5b), and values can be converted to viral copies/mg, for tissues and feces, or viral copies/mL, for body fluids like urine and serum [7]. According to the OIE, in a 10% liver homogenate, there are between 10^6^ and 10^9^ genome copies/mL [4]. Notably, as most RT-qPCRs target the VP60 and the assay detects both genomic and subgenomic RNA, the value obtained does not correspond to genome copies but to capsid copies [31]. 

In relative quantification, viral RNA load is not precisely calculated, but cycle threshold (Ct) values can be compared to positive/control samples; the lower the Ct value, the higher the initial viral load and thus an estimation of target copy numbers is possible [154]. Runs must include no template controls and conventional PCR and Sanger sequencing might be used to confirm doubtful results [157]. Optionally, a melt curve—generated by monitoring the fluorescence signal with small increment temperatures—is run at the end of the denaturing-annealing-extension cycles to validate the specificity of amplification products and identify the presence of primer dimer (Figure 5c).

A comparison between real-time RT-PCR and conventional RT-PCR highlights some of its advantages, namely the reduced risk of cross-contamination. To this greatly contributes the use of a one-tube protocol that avoids post-PCR handling, which in turn significantly shortens the reaction and the diagnostic times [7,156,158,159]. RNA virus degradation is less challenging as the target amplicons are usually smaller, thus also increasing sensitivity [7,158], and intra and inter-assay variability is low [154,156]. Detection limits of RHDV real-time RT-PCRs range from 9–100 copies [7,27,156,160] and have been shown to be similar to those of nested PCR [155]. Hence, real-time RT-PCR is the best choice when studying early stages of infection and when expecting low viral loads [7], which can result from sample exposure to unfavorable conditions for a long period of time, sample contamination or vaccination remnants [31]. If researchers opt for RT-qPCR, disease progression can be monitored by determining viral loads in multiple organs, and if Taqman probes are used the method has increased specificity [7,159]. On the other hand, performance of quantitative assays might be compromised when single nucleotide mismatches occur at the primer binding site, by impairing amplification leading to an underestimation of copy numbers due to shifts in Ct values [161].

In the last few years, significant findings on RHDV epidemiology relied on RT-qPCR results. For example, the quantification of RHDV replication and shedding in young rabbits showed their ability to spread the virus before seroconversion [162]. The liver, spleen and kidney were confirmed as the main replication sites as they contain the highest number of viral RNA copies [7,155]. The fecal-oral transmission route and the persistent infection of non-pathogenic lagoviruses was elucidated by RT-qPCR [27,160]. Furthermore, it allowed the detection of low amounts of GI.2 in carcasses of non-target species, such as the Mediterranean pine vole (*Microtus duodecimcostatus*) and the white-toothed shrew (*Crocidura russula*) [163]. Even acknowledging that the number of genome copies is not directly proportional to the number of infectious particles, RT-qPCR is also useful to confirm successful viral replication in inoculation trials [75,164]. Moreover, it has been shown that RHDV quantification in rabbit feces and in carrion flies can be used as sentinels in epidemiological surveys [134,165]. These are cost-effective methods to monitor outbreaks without the need of lagomorph carcasses [165], which in the wild are often rapidly removed by scavenger animals.

## 11. Next-Generation Sequencing

Apparently healthy species harbor a wealth of viruses, and hence a wide range of pathogens is being increasingly identified by next-generation sequencing (NGS) approaches. The characterization of such viruses is crucial for understanding virus evolution, changes in virulence and host jumps [166]. NGS technology allows for reliable and deep sequencing of the genome, with a fast turn-around time for large datasets in which respects to sample preparation. The ability to sequence multiple regions at once is an advantage, which is not possible in Sanger sequencing that only produces one read of <1000 bp per run. Additionally, it has a higher sensitivity than RT-PCR and might overcome RT-PCR primer issues, for example, mutations occurring in the primer annealing region of rapidly evolving viruses do not interfere with metagenomics analysis. On the other hand, when dealing with low number of samples, NGS is time-consuming and less cost-effective than the more familiar workflow of conventional RT-PCR or RT-qPCR. Due to its higher sensitivity, sample preparation is associated with a higher risk of contamination than, for example, PCR-based methods [167]. Data analysis remains a challenge for most researchers in the field and, depending on the purpose of the study and the complexity and dimension of the dataset, it might be a long-lasting process. Moreover, NGS techniques might not be able to detect mixed infections by similar strains [31], and the equipment required (or hiring of the service) is expensive. Nonetheless, the overall powerfulness of this tool explains its increasing popularity in molecular virology [138].

The protocol used for NGS of lagoviruses varies, especially in what refers to library preparation. While in some cases DNA libraries are prepared from a pool of conventional PCR amplicons [116,168,169], others use the entire cDNA of the specimens as a source [119,170] or the total sample RNA with host rRNA depletion [166]. Sonication or enzymatic digestion are used to fragment nucleic acids, and MiSeq has been the top choice system with 100–300 bp paired-end runs. After sequence data processing and assembly, cleaned reads are mapped against reference lagovirus genomes to generate consensus.

Highly pathogenic lagovirus strains are the major focus on RHDV research (either for controlling or increasing rabbit numbers), but an expanding genetic diversity concerning benign lagoviruses has been described [26,27,32,171,172,173]. Studies employing NGS further identified a multiplicity of other lagovirus genomes [116,166,174] that would remain undetected if using only routine diagnostic assays. These studies further show the potential of these benign viruses to provide a gene pool for recombination events, as two novel recombinant forms were described (GI.4*like*P-GI.1a and GII.1P-GI.2) [116,174]. Indeed, given the extent of recombination found in RHDV, e.g., [29,30,153], high-throughput sequencing methods are a valuable tool for the identification of instances of intergenotypic recombination [31]. Hence, the true diversity of the genus *Lagovirus* is still likely underestimated, highlighting the importance of whole genome sequencing in this field [166,174].

## 12. Lateral Flow Immunoassay

The main drawback of traditional laboratory diagnostics, either WB, ELISA, PCR or others, is the time they require to obtain a complete analysis and a diagnostic result. Furthermore, they often require trained personnel and costly equipment. This is of special relevance for rabbit farmers, who need to act quickly when facing a possible outbreak, but also for countries where access to more sophisticated/advanced laboratorial equipment and specialized personnel is restricted. To overcome that, lateral flow immunochromatographic assays (LFIA) have been developed for the detection of RHDV. This type of assay relies on a chemical colored reaction on a pad that contains immobilized specific antibodies that bind to RHDV antigens. Briefly, liver homogenates or abdominal liquid exudates diluted in specific buffers are added to the wells and move upwards on the membrane by capillary action. When they hit the conjugation pad that contains anti-RHDV MAbs, any RHDV antigens present will adhere to the membrane and produce a visible, colored line. Samples will continue to migrate towards a control label solution, where a colored line appears in order to validate the test. Dalton et al. developed a LFIA test specific for GI.2, with a detection limit of 7.8 ng/mL [175], and commercial kits might be soon available.

Although LFIAs are quite rapid, producing results in about 10 min, and do not require any specialized equipment and technical staff, they are not designed for analyzing multiple samples simultaneously and they are not able to quantify the amount of viral particles present in the sample [176]. They are also less sensitive than other methods such as RT-PCR or ELISA [175]. Thus, when using this type of test, other complementary diagnostic tools should follow to confirm the results. However, LFIA is a user-friendly test, meaning that rabbit farmers might be able to apply it, interpret the results and act rapidly to contain possible outbreaks. Overall, it has great potential as a diagnostic test to be applied both in the field and in rabbit production systems and should be taken into account as an alternative for more specialized diagnostic tools.

## 13. Luminex xTAG and xMAP Assays

**Figure 6:**
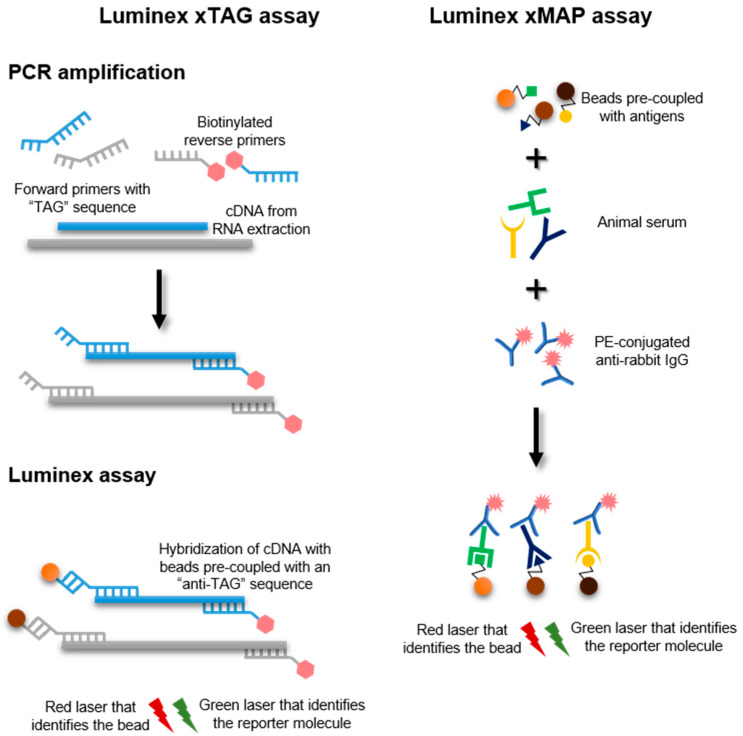
Schematic diagram of the Luminex xTAG and xMAP assays.

Rabbits are infected by a multitude of pathogens, and the investigations of possible causes of death might not be straightforward. Likewise, antibody detection against these pathogens requires separate testing for each pathogen, which complicates serosurveillance in rabbitries and monitoring of laboratory animals. Luminex technology allows to simultaneously detect numerous antigens or antibodies in a single reaction. A high-throughput Luminex xTAG assay that detects RHDV, among other pathogens, was recently developed, though discrimination of RHDV genotypes and variants is not possible [177]. Briefly, this multiplex assay has specific primers for each pathogen: a unique “TAG” modified forward primer and a biotinylated reverse primer (Figure 6). A multiplex PCR amplification is performed as in conventional RT-PCR, followed by coupling with an anti-TAG magnetic fluorescent microsphere and streptavidin-phycoerythrin conjugate. The former is recognized by a red laser in a Luminex instrument, while a green reporter laser detects the amount of labeled target, providing a quantitative readout [178]. RHDV detection with Luminex xTAG assay is specific and sensitive (detection limit of 100 copies/µL) [177]. This approach can integrate up to 500 target pathogens, and may represent a good alternative to more traditional nucleic acid-based methods, although it requires a specialized equipment.

A Luminex xMAP assay has also been developed, allowing a specific detection of anti-RHDV antibodies [179]. In this case, antigens coupled with fluorescent magnetic beads are incubated with rabbit serum and transferred to a microplate (Figure 6). The Luminex reader detects fluorescence emitted by a phycoerythrin-conjugated goat anti-rabbit IgG [179]. Comparison with commercial ELISA kits showed similar sensitivity, and thus the potential of Luminex xMAP assay as an alternative for more labor-intensive methods deserves attention in the near future. However, higher costs and the need for specialized staff are associated with this assay.

## 14. *Staphylococcus* Protein A Coagglutination Test 

**Figure 7:**
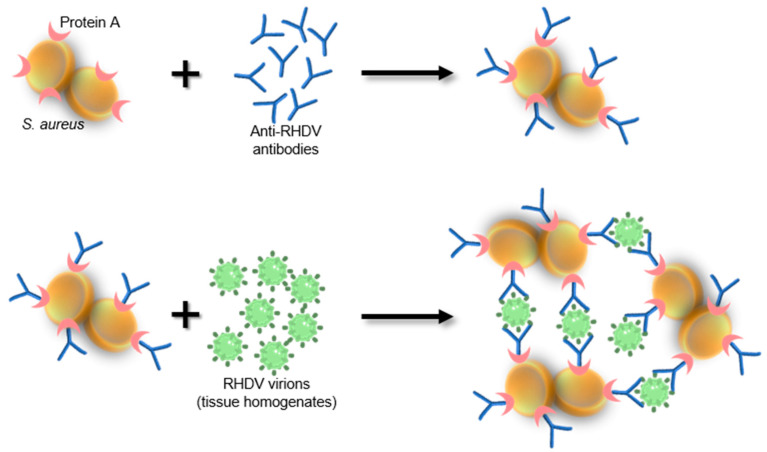
Schematic diagram of the Sp A COAT test.

The *Staphylococcus* protein A coagglutination test (Sp A COAT) is a simple, specific and sensitive test that was developed as a rapid test for the detection of RHDV [180]. The test was adapted from a slide-agglutination method for pneumococci typing based on the ability of the Fc fragments of anti-pneumococci IgG to bind to the *Staphylococcus aureus* protein-A (Sp A) located in the cell wall [181]. This binding directs the Fab structures outwards and coagglutination occurs when a suspension of homologous pneumococci antigens is added [181]. Liver, spleen, kidney and lung samples were successfully used for RHDV antigen detection by Sp A COAT with *Staphylococcus aureus* sensitized with rabbit anti-RHDV antibodies (Figure 7) [180]. The test revealed high specificity and sensitivity and better performance than the HA [180]. However, and while the Sp A COAT does not require any special equipment and provides fast results [180], it does require rabbits to be infected with RHDV in order to obtain anti-RHDV antibodies raising concerns in terms of animal welfare and it does not allow genotype or variant identification.

## 15. Experimental Infections

The lack of an appropriate cell culture system for RHDV has been a major obstacle in the study of RHDV in terms of host entry and replication [1]. Hence, some aspects of virus characterization depend on experimental infection of rabbits, which has been also used for diagnostic purposes. Despite the animal ethical concerns and the biological risk associated, rabbit inoculation trials have significantly contributed to our knowledge on RHD and RHDV by allowing, for example, the establishment of the pathogenicity of emerging variants [182], the evaluation of cross-protection between genotypes and genogroups [64,123,183], the production of commercially available vaccines from tissues of experimentally infected rabbits [1], and the determination of the expected clinical progress of the disease, considering the different variants and genotypes [4]. Experimental infections should not be regarded as a routine diagnostic method and their need for obtaining insights into the disease and the virus must be carefully and thoroughly determined before conducting them.

## 16. Conclusions

Despite circulating for more than 35 years, RHDV still takes a significant economic and ecological toll. When dealing with suspected RHDV cases, researchers shall be aware of the biological risks associated with the manipulation of potential infectious viral particles, not only for their own safety (even if there is no evidence of zoonotic risk) but also for the environment. Biosafety measures and trained laboratory staff are thus essential to prevent anthropogenic-mediated transmission of the virus.

The Reference Laboratory on RHDV currently proposes standardized diagnostic tests for pathogenic rabbit lagoviruses [4], but for countries/regions experiencing RHD outbreaks for the first time, it might be difficult to decide on which test to use. Indeed, laboratories worldwide doing research on RHD and RHDV often use their own in-house virological and serological tests developed based on their expertise, workload, budget, aims, etc. For high-throughput screening, the most cost-effective methods might include ELISA, RT-PCR, RT-qPCR and NGS.

The rapidly evolving nature of lagoviruses requires a constant re-evaluation and adaptation of detection methods. In addition, their diversity and affected leporid hosts remains largely undeciphered, adding complexity to their detection. With this review, we hope to have contributed to an informed discussion on the methods currently used that can aid in providing timely responses for disease management (Table 1). While high-throughput sequencing has shown potential to improve our knowledge on lagoviruses diversity and contribute to their surveillance [116], it might not be universally accessible. Instead, an international validated diagnostic protocol might result from a combination of simpler, yet standardized techniques.

## Figures and Tables

**Table 1:** Characteristics of the most commonly used methods for detection of rabbit hemorrhagic disease virus (RHDV).

Target	Type of Assay	Type of Sample	Detection Limit	Ability to Distinguish Variants and Genotypes	Application Scope
RHDV particles	HA	Liver, spleen, serum	n.d.	No	Virus detection
	EM	Liver, lung, kidney, spleen	10^5^ viral particles/mL	No	Virus detection, localization, distribution, VLP correct self-assembly
RHDV antigens	IHC	Liver ^1^	10^7^–10^8^ viral copies/mg	Yes ^2,3^	Virus detection, localization, distribution
	ELISA-Ag	Liver, spleen, blood, serum, heart	<HA	Yes ^2,3^	Virus detection
	LFIA	Liver, abdominal liquid exsudate	7.8 ng/mL	Yes ^2^	Virus detection
	Sp A COAT	Liver, spleen, kidney, lung	<HA	No	Virus detection
RHDV RNA	ISH	Liver, duodenum ^4^	<IHC	Yes ^2,3^	Virus detection, replication sites
	RT-PCR	Liver, kidney, spleen, lung, heart, brain, bone marrow, muscle, spinal cord, thymus, lymph, nasal secretions, urine, feces, sera	12 copies	Yes ^2,3^	Virus detection, genome characterization ^5^
	RT-qPCR	Same as RT-PCR	9 copies	Yes ^2,3^	Virus detection, genome characterization ^5^
	NGS	Liver ^1^	n.d.	Yes	Virus detection, genome characterization, virus differentiation
	Luminex xTAG	Liver ^1^	100 copies/µL	No	Virus detection
RHDV capsid proteins	WB	Liver	n.d.	Yes ^2,3^	Virus detection, validation of VLPs size
Anti-RHDV antibodies	WB	Serum	n.a.	No	Serosurveillance; mapping epitopes, confirming MAbs specificity and suitability
	HIT		n.a.	No	Serosurveillance
	ELISA-Ab		n.a.	Yes ^2,3^	Serosurveillance
	Luminex xMAP		n.a.	No	Serosurveillance

^1^ Other tissues might be used; ^2^ Genotypes, depending on the MAb/probe/primers used; ^3^ Variants, depending on the MAb/primers used; ^4^ For GI.4 strains; ^5^ If followed by Sanger sequencing.

## Data Availability

Not applicable.
